# Exact non-static cylindrical solutions of Einstein’s field equations with imperfect fluids

**DOI:** 10.1371/journal.pone.0355505

**Published:** 2026-08-31

**Authors:** Tahir Hussain, Muhammad Farhan, Hala Mostafa, Sk. A. Shezan, Nowshad Amin, Rayan Hamza Alsisi, Naveed Ahmad

**Affiliations:** 1 Department of Mathematics, University of Peshawar, Khyber Pakhtunkhwa, Pakistan; 2 School of Energy and Power Engineering, Shandong University, Jinan, People’s Republic of China; 3 Department of Information Technology, College of Computer and Information Sciences, Princess Nourah bint Abdulrahman University, Riyadh 11671, Saudi Arabia; 4 Department of Electrical Engineering, Prince Faisal bin Khalid bin Sultan Research Chair in Renewable Energy Studies and Applications (PFCRE), Northern Border University, Arar, Saudi Arabia; 5 Department of Electrical Engineering, Faculty of Engineering, Islamic University of Madinah, Madinah 41411, Saudi Arabia; 6 Department of Chemical and Materials Engineering, College of Engineering, Northern Border University, Arar, Saudi Arabia; Yibin University, CHINA

## Abstract

This work systematically derives a broad class of exact cylindrically symmetric spacetime models possessing scaling symmetries of varying dimensions. These models are then used to solve Einstein’s field equations for an imperfect fluid source that yields explicit anisotropic energy-momentum distributions. The solutions include vacuum, anisotropic fluid, and wave-like geometries, with connections to known models such as cylindrical waves, Levi-Civita spacetimes, and Kasner-type expansions. This study extends the catalog of exact cylindrically symmetric solutions and illustrates how scaling symmetry organizes the physics of time-dependent, anisotropic spacetimes.

## 1. Introduction

In the framework of the general theory of relativity, symmetries of spacetimes are of particular importance, as they offer profound insight into both the physical nature of gravitational fields and the underlying mathematical structure of Einstein’s field equations. Spacetime symmetries are defined through transformations that preserve some physical or geometric features of spacetimes and therefore play an important role in understanding their behavior. In particular, the symmetries of the metric tensor, namely Killing, homothetic and conformal symmetries occupy a prominent position due to their wide applicability and physical relevance. The presence of these symmetries significantly reduces the complexity of the governing equations of general relativity, the Einstein’s field equations (EFEs), thereby facilitating the search for exact and physically meaningful solutions. In addition to their role in finding the exact solutions of EFEs and their classification, these symmetries are closely connected with conservation laws and invariance properties of spacetime. Moreover, spacetime symmetries contribute to a deeper understanding of the geometric, causal, and structural aspects of spacetime, helping to clarify how matter, energy, and geometry are interconnected within the theory of general relativity.

Spacetime symmetries are described by vector fields that characterize how the metric tensor behaves under continuous transformations. A vector field *V* is known as a Killing vector field (KVF) if it generates an isometry of the spacetime. Mathematically, this condition is expressed by the vanishing of the Lie derivative of the metric tensor along *V*, that is ${ℒ}_{V}g_{ab}=0$ [1]. This condition implies that the metric remains invariant under the associated transformation, leading to the preservation of distances and angles. A homothetic vector field (HVF) generalizes the concept of a KVF by allowing the metric to change by a constant scaling factor. In such a case, the Lie derivative of $g_{ab}$ is proportional to $g_{ab}$ itself with a constant coefficient, representing a uniform self-similarity of spacetime, that is ${ℒ}_{V}g_{ab}=2cg_{ab},$ where *c* is a constant. Similarly, a conformal vector field satisfies the relation ${ℒ}_{V}g_{ab}=2\psi (x^{a})g_{ab},$ with $\psi (x^{a})$ being a smooth function of the spacetime coordinates. These symmetries preserve the metric up to a local scale factor and include Killing and homothetic symmetries as special cases. Conformal symmetries are fundamental in the study of spacetime geometry and play an important role in simplifying EFEs and revealing their underlying geometric structure.

In literature, various spacetimes are classified through the above defined spacetime symmetries [2–12]. However, most of these studies have focused on static or simpler spacetime geometries because such geometries have easier mathematical structure and the corresponding governing equations representing spacetime symmetries can be solved more conveniently. On the other hand, non-static spacetime geometries have received comparatively less attention. Due to the time-dependent nature of non-static spacetimes, the governing equations become more difficult to analyze. Consequently, exploring symmetry properties in such spacetimes and finding the exact solutions remains a challenging task. Therefore, non-static spacetime geometries are still not as well explored as their static counterparts.

Furthermore, most of the available studies on spacetime symmetries are primarily focused only on the mathematical analysis. The physical importance of the spacetime metrics derived during a symmetry classification is often not investigated with sufficient details. Particularly, the derived spacetime models are rarely examined as exact solutions of EFEs for some specific forms of the stress-energy tensor. Consequently, the physical viability of the derived spacetime metrics remain largely unexplored.

In the present study, we aim to investigate HVFs of non-static cylindrically symmetric spacetime and solve the EFEs for the resulting metrics for an imperfect fluid source. Cylindrical symmetry plays a pivotal role in the theory general relativity. This symmetry arises in the study of many physically relevant systems such as cosmic strings, gravitational waves and anisotropic matter distributions. The non-static nature of the spacetime allows the investigation of time-dependent gravitational fields, which are essential for understanding realistic astrophysical and cosmological processes. Moreover, the combination of cylindrical symmetry with temporal evolution provides a rich geometric structure that is sufficiently general to capture interesting physical behavior, while still allowing for meaningful analytical treatment. For these reasons, non-static cylindrically symmetric spacetime serves as a suitable and physically motivated framework for the present investigation.

In order to classify a spacetime through the above defined spacetime symmetries, one requires to solve a system of partial differential equations generated by the underlying symmetry conditions. Traditionally, such systems of partial differential equations are solved using the method of direct integration. In this technique, the coupled system of equations representing the underlying symmetry is first decoupled and then integrated to derive the explicit forms of the symmetry vector fields. The procedure typically leads to various distinct cases, depending upon the conditions on the metric coefficients for which the considered spacetime admits the desired symmetries. However, this approach is often observed to be cumbersome, and in practice it may overlook potentially significant spacetime metrics possessing the required symmetry properties.

On the other hand, the recently introduced Rif tree approach has emerged as a powerful tool for the classification of spacetimes. This approach is based on a computer algorithm (Rif algorithm), implemented in Maple, which transforms the system of symmetry equations into an involutive form, enabling their systematic solution and facilitating the classification of vector fields, such as HVFs. The algorithm starts with calling the “Exterior” package in Maple with the command “with(Exterior).” The second step is to introduce the system of symmetry equations using the command “sysDEs.” Once the symmetry equations are inserted, the symmetry command “eq: = findsymmetry” is used to analyze the symmetry equations and to determine the constraints on the metric coefficients under which the desired symmetry vector fields exist. As a result, the conditions on the metric coefficients are explicitly displayed that typically involve the derivatives of these coefficients. In this way, the problem of solving the symmetry equations splits into different cases and a graphical visualization of these cases is obtained using the command “caseplot(eq, pivots).” This graphical visualization is displayed as a tree-like structure, called the Rif tree. Each branch of the Rif tree corresponds to different set of constraints for which the considered metric may admit the desired symmetries. The final step is to solve the symmetry equations separately along each branch, giving the explicit forms of the desired symmetries.

The main advantage of this recently introduced approach is that it provides a more systematic and comprehensive classification of spacetimes via their symmetries. In recent literature, this approach has been successfully used to classify different spacetime symmetries, and as a result additional spacetime metrics have been identified that remained inaccessible using the traditional direct integration method [13–21]. In this paper, we use Rif tree approach for finding HVFs of non-static cylindrically symmetric spacetime. The aim is to find all non-static cylindrically symmetric spacetime metrics possessing proper HVF and to solve the EFEs for the resulting metrics for an imperfect fluid source. To the best of our knowledge, there is no available study in literature which explicitly constructs the imperfect fluid solutions of the EFEs by employing a complete Rif tree classification of HVFs in non-static cylindrical spacetimes. The present analysis aims to fill this gap and provides a unified catalog of exact anisotropic cylindrical spacetimes with proper HVFs.

The motivation behind adopting the Rif tree approach for the current analysis is the highly overdetermined nature of the symmetry equations arising from the definition of HVFs. Unlike the conventional direct integration method, this approach systematically decomposes the symmetry equations into compatible branches and ensures that all admissible solution classes are identified. In this way, the possibility of overlooking nontrivial symmetry structures is significantly reduced and a complete classification of the considered spacetime is achieved. Consequently, the method serves as a framework for obtaining a complete classification of homothetic symmetries.

The paper is organized as follows. In section 2, we study the homothetic symmetries of the considered spacetime using Rif tree method. The EFEs with an imperfect fluid source are solved for the obtained metrics in section 3. Section 4 presents a connection of the derived metrics with the existing known solutions in the literature. The conclusion of the present study is given in the last section.

## 2. Homothetic symmetries

We consider the following metric of non-static cylindrically symmetric spacetime [22]:


$$\begin{array}{c} ds^{2}=-F^{2}(t,r)\;dt^{2}+dr^{2}+G^{2}(t,r)d\theta^{2}+H^{2}(t,r)dz^{2}. \end{array}$$ (2.1)

Here *r* denotes the radial distance from the symmetry axis, θ is the angular coordinate around the axis such that θ∈[0,2π] and z∈R is the coordinate along the axis of symmetry. This metric admits two independent KVFs, given by $\xi_{\left(1\right)}=\partial_{\theta }$ and $\xi_{\left(2\right)}=\partial_{z}.$ If the metric coefficients *F*, *G*, and *H* depend only on *r*, this metric represents static cylindrically symmetric spacetime possessing an additional Killing symmetry $\partial_{t}.$ We use the condition ${ℒ}_{V}g_{ab}=2cg_{ab},$ satisfied by homothetic vector fields, for the above metric to get the following system of partial differential equations.


$$\begin{array}{c} F_{,t}\;V^{0}+F_{,r}\;V^{1}+F\;V_{,t}^{0}=c\;F \end{array}$$ (2.2)

$$\begin{array}{c} V_{,r}^{1}=c \end{array}$$ (2.3)

$$\begin{array}{c} G_{,t}\;V^{0}+G_{,r}\;V^{1}+G\;V_{,\theta }^{2}=c\;G \end{array}$$ (2.4)

$$\begin{array}{c} H_{,t}\;V^{0}+H_{,r}\;V^{1}+H\;V_{,z}^{3}=c\;H \end{array}$$ (2.5)

$$\begin{array}{c} F^{2}\;V_{,r}^{0}-V_{,t}^{1}=0 \end{array}$$ (2.6)

$$\begin{array}{c} F^{2}\;V_{,\theta }^{0}-G^{2}\;V_{,t}^{2}=0 \end{array}$$ (2.7)

$$\begin{array}{c} F^{2}\;V_{,z}^{0}-H^{2}\;V_{,t}^{3}=0 \end{array}$$ (2.8)

$$\begin{array}{c} V_{,\theta }^{1}+G^{2}\;V_{,r}^{2}=0 \end{array}$$ (2.9)

$$\begin{array}{c} V_{,z}^{1}+H^{2}\;V_{,r}^{3}=0 \end{array}$$ (2.10)

$$\begin{array}{c} G^{2}\;V_{,z}^{2}+H^{2}\;V_{,\theta }^{3}=0 \end{array}$$ (2.11)

**Fig 1:**
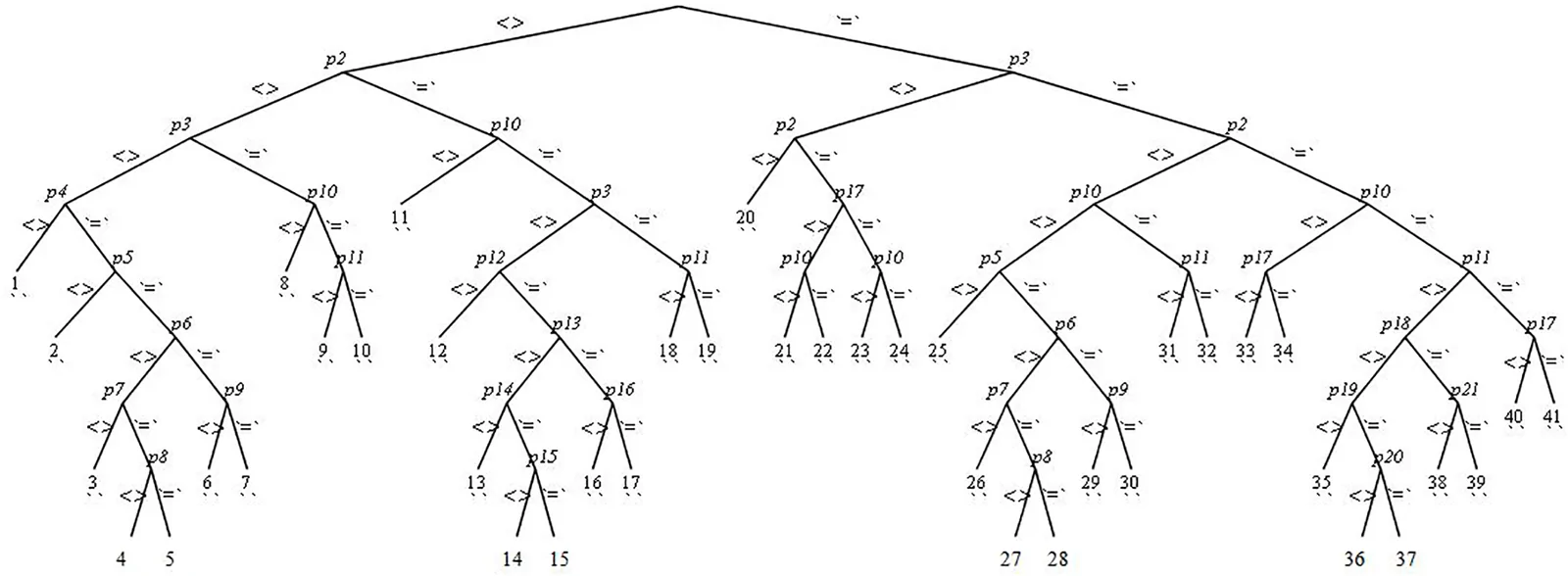
Rif Tree.

The constant *c* appearing in the above equations play an important role in distinguishing proper HVFs from KVFs. For *c* = 0, the vector field *V* in the above equations represents a KVF which generates an isometry, preserving all distances. When c≠0, the vector field *V* denotes a proper HVF. The magnitude of the constant *c* controls the overall rate of uniform scaling. The above symmetry equations are examined using Rif algorithm to restrict the functions *F*, *G* and *H* such that the metric (2.1) possesses proper HVFs. As a result, we have obtained the Rif tree, given in Fig 1, along with its nodes as defined in (2).


$$\begin{array}{cc} p1 & =G_{,r}\\ p2 & =H_{,r}\\ p3 & =G_{,t}\\ p4 & =G_{,r}\;H_{,t}-G_{,t}\;H_{,r}\\ p5 & =F_{,r}\;H_{,t}-F_{,t}\;H_{,r}\\ p6 & =H_{,tr}\;H_{,t}-H_{,tt}\;H_{,r}\\ p7 & =(H_{,t}{)}^{2}\;H_{,rr}-2H_{,tr}\;H_{,t}\;H_{,r}+H_{,tt}\;(H_{,r}{)}^{2}\\ p8 & =H_{,ttt}\;H_{,t}\;H_{,r}+2H_{,tr}\;H_{,t}\;H_{,tt}-2(H_{,tt}{)}^{2}\;H_{,r}-H_{,ttr}\;(H_{,t}{)}^{2}\\ p9 & =H_{,rr}\;(H_{,t}{)}^{2}-H_{,tt}\;(H_{,r}{)}^{2}\\ p10 & =H_{,t}\\ p11 & =F_{,t}\\ p12 & =F_{,r}\;G_{,t}-F_{,t}\;G_{,r}\\ p13 & =G_{,tr}\;G_{,t}-G_{,tt}\;G_{,r}\\ p14 & =(G_{,t}{)}^{2}\;G_{,rr}-2G_{,tr}\;G_{,t}\;G_{,r}+G_{,tt}\;(G_{,r}{)}^{2}\\ p15 & =G_{,ttt}\;G_{,t}\;G_{,r}+2G_{,tr}\;G_{,tt}\;G_{,t}-2(G_{,tt}{)}^{2}\;G_{,r}-G_{,ttr}\;(G_{,t}{)}^{2}\\ p16 & =G_{,rr}\;(G_{,t}{)}^{2}-G_{,tt}\;(G_{,r}{)}^{2}\\ p17 & =F_{,r}\\ p18 & =F_{,tr}\;F_{,t}-F_{,tt}\;F_{,r} \end{array}$$

$$\begin{array}{cc} p19 & =(F_{,t}{)}^{2}\;F_{,rr}-2F_{,tr}\;F_{,t}\;F_{,r}+F_{,tt}\;(F_{,r}{)}^{2}\\ p20 & =F_{,ttt}\;F_{,t}\;F_{,r}+2F_{,tr}\;F_{,tt}\;F_{,t}-2(F_{,tt}{)}^{2}\;F_{,r}-F_{,ttr}\;(F_{,t}{)}^{2}\\ p21 & =F_{,rr}\;(F_{,t}{)}^{2}-F_{,tt}\;(F_{,r}{)}^{2} \end{array}$$ (2.12)

In the Rif tree, each pivot $p_{i}$ splits into two cases, that is $p_{i}=0$ and $p_{i}\text{≠}0.$ The path from the root to a leaf gives rise to a branch, that imposes some conditions on the metric coefficients. These conditions are used to solve equations (2.2)–(2.11), yielding the resulting specific metrics and their corresponding symmetries. The derived metrics along with their proper HVFs and additional KVFs are presented in Tables 1–4. The metrics with only three symmetries (one proper HVF and two KVFs) are labeled by 3a−3l. The metrics 4a−4g admit one proper HVF and three KVFs, while those labeled by 5a−5k possess four KVFs along with one proper HVF. Finally, each of the metrics 11a−11i possesses one proper HVF and ten KVFs. In each case, $\xi_{\left(3\right)}$ denotes a proper HVF, while other $\xi_{\left(i\right)}$s are additional KVFs.

**Table 1:** Classification of HVFs.

Metric No./ Branch No.	Metric Coefficients	Proper HVF / Additional KVFs
3a 10	*F* = *const*. $G=(a_{1}r+a_{2}{)}^{1-\frac{a_{3}}{a_{1}}},$ $H=(a_{1}t+a_{4}{)}^{1-\frac{a_{5}}{a_{1}}}$ where $a_{1}\text{≠}0$, $a_{1}\text{≠}a_{3}$ and $a_{1}\text{≠}a_{5}$	$\xi_{\left(3\right)}=t\partial_{t}+r\partial_{r}$
3b 18	$F=H=a_{1}r+a_{2},$ *G* = *G*(*t*), where $a_{1}\text{≠}0$ and $G^{\prime }(t)\text{≠}0$	$\xi_{\left(3\right)}=\frac{a_{1}r+a_{2}}{a_{1}}\partial_{r}+\theta \partial_{\theta }$
3c 18	$F=a_{1}r+a_{2}$, *G* = *G*(*t*), $H=(a_{1}r+a_{2}{)}^{1-\frac{a_{3}}{a_{4}}}$ where $G^{\prime }(t)\text{≠}0,$ $a_{1}\text{≠}0$, $a_{4}\text{≠}0$ and $a_{4}\text{≠}a_{3}$	$\xi_{\left(3\right)}=\frac{a_{1}r+a_{2}}{a_{1}}\partial_{r}+\theta \partial_{\theta }$
3d 18	$F=(a_{5}r+a_{6}{)}^{1-\frac{a_{3}(a_{1}-a_{2})}{a_{1}}}$, $G=(a_{3}t+a_{4}{)}^{\frac{1}{a_{3}}}$, $H=a_{5}r+a_{6}$ where $a_{1}\text{≠}0$, $a_{3}\text{≠}0$, $a_{5}\text{≠}0$, $a_{1}\text{≠}a_{2}$ and $a_{1}\text{≠}(a_{1}-a_{2})a_{3}$	$\xi_{\left(3\right)}=(a_{3}t+a_{4})\partial_{t}+\frac{a_{5}r+a_{6}}{a_{5}}\partial_{r}$
3e 18	$F=(a_{1}r+a_{2}{)}^{1-\frac{a_{3}(a_{1}-a_{4})}{a_{1}}}$, $G=(a_{3}t+a_{5}{)}^{\frac{1}{a_{3}}}$, $H=(a_{1}r+a_{2}{)}^{1-\frac{a_{6}}{a_{1}}}$ where $a_{1}\text{≠}0$, $a_{3}\text{≠}0$, $a_{1}\text{≠}a_{4}$, $a_{1}\text{≠}a_{6}$ and $a_{1}\text{≠}a_{3}(a_{1}-a_{4})$	$\xi_{\left(3\right)}=(a_{3}t+a_{5})\partial_{t}+r\partial_{r}$
3f 19	$F=(a_{1}r+a_{2}{)}^{1-\frac{a_{3}}{a_{1}}},$ $G=(a_{4}t+a_{5}{)}^{\frac{a_{1}-a_{6}}{a_{4}}}$, $H=(a_{4}t+a_{5}{)}^{\frac{a_{1}-a_{7}}{a_{4}}}$ where $a_{1}\text{≠}0$, $a_{4}\text{≠}0$, $a_{1}\text{≠}a_{3}$, $a_{1}\text{≠}a_{6}$ and $a_{1}\text{≠}a_{7}$	$\xi_{\left(3\right)}=r\partial_{r}$
3g 21	*F* = *const*., $G=(a_{1}t+a_{2}{)}^{1-\frac{a_{3}}{a_{1}}},$ $H=(a_{1}r+a_{4}{)}^{1-\frac{a_{5}}{a_{1}}}$ where $a_{1}\text{≠}0$, $a_{1}\text{≠}a_{3}$ and $a_{1}\text{≠}a_{5}$	$\xi_{\left(3\right)}=t\partial_{t}+r\partial_{r}$
3h 20	$F=a_{1}r+a_{2}$, *G* = *G*(*t*), *H* = *const*. where $a_{1}\text{≠}0$ and $G^{\prime }(t)\text{≠}0$	$\xi_{\left(3\right)}=\theta \partial_{\theta }+z\partial_{z}$
3i 20	$F=(a_{1}r+a_{2}{)}^{1-\frac{a_{3}}{a_{1}}}$, $G=(a_{4}t+a_{5}{)}^{\frac{a_{1}-a_{6}}{a_{4}}}$, *H* = *const*., where $a_{1}\text{≠}0$, $a_{4}\text{≠}0$, $a_{1}\text{≠}a_{3}$ and $a_{1}\text{≠}a_{6}$	$\xi_{\left(3\right)}=r\partial_{r}+z\partial_{z}$
3j 31	$F=(a_{1}r+a_{2}{)}^{1-a_{3}},$ *G* = *const*., $H=(a_{3}t+a_{5}{)}^{\frac{1}{a_{3}}}$ where $a_{1}\text{≠}0$ and $a_{3}\text{≠}0$	$\xi_{\left(3\right)}=(a_{3}t+a_{5})\partial_{t}+r\partial_{r}+\theta \partial_{\theta }$
3k 31	$F=a_{1}r+a_{2},$ *G* = *const*., *H* = *H*(*t*) where $a_{1}\text{≠}0$, $H^{\prime }(t)\text{≠}0$ and $H(t)H^{\prime \prime }(t)-H^{\prime }(t{)}^{2}\text{≠}0$	$\xi_{\left(3\right)}=\frac{a_{1}r+a_{2}}{a_{1}}\partial_{r}+\theta \partial_{\theta }+z\partial_{z}$
3l 10	*F* = *const*., $G=(a_{1}r+a_{2}{)}^{1-\frac{a_{3}}{a_{1}}},$ $H=a_{4}t+a_{5}$ where $a_{1}\text{≠}0,$ $a_{4}\text{≠}0$ and $a_{1}\text{≠}a_{3}$	$\xi_{\left(3\right)}=\frac{a_{4}t+a_{5}}{a_{4}}\partial_{t}+r\partial_{r}$
4a 18	$F=H=a_{1}r+a_{2}$, $G=e^{t},$ where $a_{1}\text{≠}0$	$\xi_{\left(3\right)}=\partial_{t}+\frac{a_{1}r+a_{2}}{a_{1}}\partial_{r}$
		$\xi_{\left(4\right)}=-\partial_{t}+\theta \partial_{\theta }$
4b 19	$F=(a_{1}r+a_{2}{)}^{1-\frac{a_{3}}{a_{1}}},$ $G=H=(a_{3}t+a_{4}{)}^{\frac{a_{1}-a_{5}}{a_{3}}}$ where $a_{1}\text{≠}0,a_{3}\text{≠}0,\;a_{1}\text{≠}a_{3}$ and $a_{1}\text{≠}a_{5}$	$\xi_{\left(3\right)}=r\partial_{r}$
		$\xi_{\left(4\right)}=z\partial_{\theta }-\theta \partial_{z}$
4c 19	$F=a_{1}r+a_{2},$ $G=H=e^{\frac{(a_{3}-a_{4})t}{a_{5}}}$ where $a_{3}\text{≠}a_{4},$ $a_{1}\text{≠}0$ and $a_{5}\text{≠}0$	$\xi_{\left(3\right)}=\frac{a_{1}r+a_{2}}{a_{1}}\partial_{r}$
		$\xi_{\left(4\right)}=z\partial_{\theta }-\theta \partial_{z}$
4d 19	$F=\sqrt{2a_{1}r+2a_{2}},$ $G=H=(a_{3}t+4a_{4}{)}^{2-\frac{4a_{5}}{a_{3}}}$ where $a_{3}\text{≠}2a_{5},$ $a_{1}\text{≠}0$ and $a_{3}\text{≠}0$	$\xi_{\left(3\right)}=\frac{t}{2}\partial_{t}+\frac{F}{2F_{,r}}\partial_{r}$
		$\xi_{\left(4\right)}=z\partial_{\theta }-\theta \partial_{z}$
4e 9	*F* = *const*., $G=a_{1}r+a_{2},$ $H={\left(\frac{a_{3}(a_{1}r+a_{2})}{a_{1}}\right)}^{1-\frac{2a_{4}}{a_{3}}}$ where $a_{1}\text{≠}0,$ $a_{3}\text{≠}0$ and $a_{3}\text{≠}2a_{4}$	$\xi_{\left(3\right)}=t\partial_{t}+\frac{a_{1}r+a_{2}}{a_{1}}\partial_{r}$ $\xi_{\left(4\right)}=\partial_{t}$

**Table 2:** Classification of HVFs.

Metric No./ Branch No.	Metric Coefficients	Proper HVF / Additional KVFs
4f 20	$F=a_{1}r+a_{2},$ $G=e^{t},$ *H* = *const*. where $a_{1}\text{≠}0$	$\xi_{\left(3\right)}=\partial_{t}+\frac{a_{1}r+a_{2}}{a_{1}}\partial_{r}+z\partial_{z}$
		$\xi_{\left(4\right)}=-\partial_{t}+\theta \partial_{\theta }$
4g 23	*F* = *H* = *const*., $G=(a_{1}t+2a_{2}{)}^{1-\frac{2a_{3}}{a_{1}}},$ where $a_{1}\text{≠}0$ and $a_{1}\text{≠}2a_{3}$	$\xi_{\left(3\right)}=t\partial_{t}+r\partial_{r}+z\partial_{z}$
		$\xi_{\left(4\right)}=\partial_{r}$
5a 31	$F=a_{1}r+a_{2},$ *G* = *const*., $H=e^{t}$ where $a_{1}\text{≠}0$	$\xi_{\left(3\right)}=\partial_{t}+\frac{a_{1}r+a_{2}}{a_{1}}\partial_{r}+\theta \partial_{\theta }$
		$\xi_{\left(4\right)}=-\partial_{t}+z\partial_{z}$
		$\xi_{\left(5\right)}=\partial_{r}$
5b 10	*F* = *const*., $G=a_{1}r+a_{2},$ $H=(a_{3}t+2a_{4}{)}^{1-\frac{2a_{5}}{a_{3}}}$ where $a_{1}\text{≠}0,$ $a_{3}\text{≠}0$ and $a_{3}\text{≠}2a_{5}$	$\xi_{\left(3\right)}=t\partial_{t}+\frac{a_{1}r+a_{2}}{a_{1}}\partial_{r}$
		$\xi_{\left(4\right)}=a_{1}\sin(a_{1}\theta )\partial_{r}+\frac{a_{1}\cos(a_{1}\theta )}{a_{1}r+a_{2}}\partial_{\theta }$
		$\xi_{\left(5\right)}=a_{1}\cos(a_{1}\theta )\partial_{r}-\frac{a_{1}\sin(a_{1}\theta )}{a_{1}r+a_{2}}\partial_{\theta }$
5c 11	*F* = *F*(*t*), $G=(a_{1}t+2a_{2}{)}^{1-\frac{2a_{3}}{a_{1}}},$ *H* = *const*. where $F_{,t}(t)\text{≠}0,$ $a_{1}\text{≠}0$ and $a_{1}\text{≠}2a_{3}$	$\xi_{\left(3\right)}=\frac{\int F(t)dt}{F(t)}\partial_{t}+r\partial_{r}+z\partial_{z}$
		$\xi_{\left(4\right)}=\frac{z}{F(t)}\partial_{t}+\int F(t)dt\partial_{z}$
		$\xi_{\left(5\right)}=\frac{1}{F(t)}\partial_{t}$
5d 17	*F* = *H* = *const*., $G=(a_{1}t+2a_{2}{)}^{1-\frac{2a_{3}}{a_{1}}},$ where $a_{1}\text{≠}0$ and $a_{1}\text{≠}2a_{3}$	$\xi_{\left(3\right)}=t\partial_{t}+r\partial_{r}+z\partial_{z}$
		$\xi_{\left(4\right)}=z\partial_{t}+t\partial_{z}$
		$\xi_{\left(5\right)}=\partial_{r}$
5e 21	*F* = *const*., $G=a_{1}t+a_{2},$ $H=(a_{3}r+2a_{4}{)}^{1-\frac{2a_{5}}{a_{3}}}$ where $a_{1}\text{≠}0,$ $a_{3}\text{≠}0$ and $a_{3}\text{≠}2a_{5}$	$\xi_{\left(3\right)}=t\partial_{t}+r\partial_{r}+\theta \partial_{\theta }+z\partial_{z}$
		$\xi_{\left(4\right)}=a_{1}e^{a_{1}\theta }\partial_{t}-\frac{a_{1}e^{a_{1}\theta }}{a_{1}t+a_{2}}\partial_{\theta }$
		$\xi_{\left(5\right)}=a_{1}e^{-a_{1}\theta }\partial_{t}-\frac{a_{1}e^{-a_{1}\theta }}{a_{1}t+a_{2}}\partial_{\theta }$
5f 24	*F* = *F*(*t*), *G* = *const*., $H=(a_{1}r+2a_{2}{)}^{1-\frac{2a_{3}}{a_{1}}},$ where $F_{,t}(t)\text{≠}0,$ $a_{1}\text{≠}0$ and $a_{1}\text{≠}2a_{3}$	$\xi_{\left(3\right)}=\frac{\int F(t)dt}{F(t)}\partial_{t}+r\partial_{r}+\theta \partial_{\theta }$
		$\xi_{\left(4\right)}=\frac{\theta }{F(t)}\partial_{t}+\int F(t)dt\partial_{\theta }$
		$\xi_{\left(5\right)}=\frac{1}{F(t)}\partial_{t}$
5g 30	*F* = *G* = *const*., $H=a_{1}r+a_{2},$ where $a_{1}\text{≠}0$	$\xi_{\left(3\right)}=t\partial_{t}+r\partial_{r}+\theta \partial_{\theta }$
		$\xi_{\left(4\right)}=\theta \partial_{t}+t\partial_{\theta }$
		$\xi_{\left(5\right)}=\partial_{t}$
5h 38	$F=(a_{1}r+2a_{2}{)}^{1-\frac{2a_{3}}{a_{1}}},$ *G* = *H* = *const*. where $a_{1}\text{≠}0$ and $a_{1}\text{≠}2a_{3}$	$\xi_{\left(3\right)}=r\partial_{r}+\theta \partial_{\theta }+z\partial_{z}$
		$\xi_{\left(4\right)}=z\partial_{\theta }-\theta \partial_{z}$
		$\xi_{\left(5\right)}=\partial_{t}$
5i 16	*F* = *H* = *const*., *G* = *a*_1_(*t* + *r*), where $a_{1}\text{≠}0$	$\xi_{\left(3\right)}=t\partial_{t}+r\partial_{r}+z\partial_{z},$
		$\xi_{\left(4\right)}=r\partial_{t}+t\partial_{r}-\theta \partial_{\theta }$
		$\xi_{\left(5\right)}=-\partial_{t}+\partial_{r}$
5j 29	*F* = *G* = *const*., *H* = *a*_1_(*t* + *r*), where $a_{1}\text{≠}0$	$\xi_{\left(3\right)}=t\partial_{t}+r\partial_{r}+\theta \partial_{\theta },$
		$\xi_{\left(4\right)}=r\partial_{t}+t\partial_{r}-z\partial_{z}$
		$\xi_{\left(5\right)}=-\partial_{t}+\partial_{r}$
5k 30	*F* = *G* = *const*., $H=a_{1}r+a_{2},$ where $a_{1}\text{≠}0$	$\xi_{\left(3\right)}=t\partial_{t}+\frac{a_{1}r+a_{2}}{a_{1}}\partial_{r}+\theta \partial_{\theta }$
		$\xi_{\left(4\right)}=\theta \partial_{t}+t\partial_{\theta }$
		$\xi_{\left(5\right)}=\partial_{t}$

**Table 3:** Classification of HVFs.

Metric No./ Branch No.	Metric Coefficients	Proper HVF / Additional KVFs
11a 10	*F* = *const*., $G=a_{1}r+a_{2},$ $H=a_{3}t+a_{4}$ where $a_{1}\text{≠}0$ and $a_{3}\text{≠}0$	$\xi_{\left(3\right)}=\frac{a_{3}t+a_{4}}{a_{3}}\partial_{t}+\frac{a_{1}r+a_{2}}{a_{1}}\partial_{r}$
		$\xi_{\left(4\right)}=a_{1}\cos(a_{1}\theta )\partial_{r}-\frac{a_{1}}{G(r)}\sin(a_{1}\theta )\partial_{\theta }$
		$\xi_{\left(5\right)}=a_{1}\sin(a_{1}\theta )\partial_{r}+\frac{a_{1}}{G(r)}\cos(a_{1}\theta )\partial_{\theta }$
		$\xi_{\left(6\right)}=e^{-a_{3}z}\partial_{t}+\frac{e^{-a_{3}z}}{H(t)}\partial_{z}$
		$\xi_{\left(7\right)}=e^{a_{3}z}\partial_{t}-\frac{e^{a_{3}z}}{H(t)}\partial_{z}$
		$\xi_{\left(8\right)}=e^{a_{3}z}[a_{3}G(r)\cos(a_{1}\theta )\partial_{t}+a_{1}H(t)\cos(a_{1}\theta )\partial_{r}-\frac{a_{1}H(t)\sin(a_{1}\theta )}{G(r)}\partial_{\theta }-\frac{G(r)\cos(a_{1}\theta )}{H(t)}\partial_{z}]$
		$\xi_{\left(9\right)}=e^{-a_{3}z}[a_{3}G(r)\cos(a_{1}\theta )\partial_{t}+a_{1}H(t)\cos(a_{1}\theta )\partial_{r}-\frac{a_{1}H(t)\sin(a_{1}\theta )}{G(r)}\partial_{\theta }+\frac{G(r)\cos(a_{1}\theta )}{H(t)}\partial_{z}$
		$\xi_{\left(10\right)}=e^{-a_{3}z}[a_{3}G(r)\sin(a_{1}\theta )\partial_{t}+a_{1}H(t)\sin(a_{1}\theta )\partial_{r}+\frac{a_{1}H(t)\cos(a_{1}\theta )}{G(r)}\partial_{\theta }+\frac{G(r)\sin(a_{1}\theta )}{H(t)}\partial_{z}]$
		$\xi_{\left(11\right)}=e^{a_{3}z}[a_{3}G(r)\sin(a_{1}\theta )\partial_{t}+a_{1}H(t)\sin(a_{1}\theta )\partial_{r}+\frac{a_{1}H(t)\cos(a_{1}\theta )}{G(r)}\partial_{\theta }-\frac{G(r)\sin(a_{1}\theta )}{H(t)}\partial_{z}]$
11b 11	*F* = *F*(*t*) $G=a_{1}r+a_{2}$ *H* = *const*. where $F_{,t}(t)\text{≠}0$ and $a_{1}\text{≠}0$	$\xi_{\left(3\right)}=\frac{\int F(t)dt}{F(t)}\;\partial_{t}+\frac{a_{1}r+a_{2}}{a_{1}}\partial_{r}+z\partial_{z}$
		$\xi_{\left(4\right)}=\frac{1}{F(t)}\;\partial_{t}$
		$\xi_{\left(5\right)}=\frac{z}{F(t)}\;\partial_{t}+\int F(t)dt\;\partial_{z}$
		$\xi_{\left(6\right)}=-z\cos(a_{1}\theta )\partial_{r}+\frac{z\sin(a_{1}\theta )}{G(r)}\partial_{\theta }+\frac{G(r)\cos(a_{1}\theta )}{a_{1}}\partial_{z}$
		$\xi_{\left(7\right)}=-z\sin(a_{1}\theta )\partial_{r}-\frac{z\cos(a_{1}\theta )}{G(r)}\partial_{\theta }+\frac{G(r)\sin(a_{1}\theta )}{a_{1}}\partial_{z}$
		$\xi_{\left(8\right)}=\cos(a_{1}\theta )\partial_{r}-\frac{\sin(a_{1}\theta )}{G(r)}\partial_{\theta }$
		$\xi_{\left(9\right)}=\sin(a_{1}\theta )\partial_{r}+\frac{\cos(a_{1}\theta )}{G(r)}\partial_{\theta }$
		$\xi_{\left(10\right)}=\frac{G(r)\cos(a_{1}\theta )}{a_{1}F(t)}\partial_{t}+\cos(a_{1}\theta )\int F(t)dt\;\partial_{r}-\frac{\sin(a_{1}\theta )\int F(t)dt}{G(r)}\partial_{\theta }$
		$\xi_{\left(11\right)}=\frac{G(r)\sin(a_{1}\theta )}{a_{1}F(t)}\partial_{t}+\sin(a_{1}\theta )\int F(t)dt\;\partial_{r}+\frac{\cos(a_{1}\theta )\int F(t)dt}{G(r)}\partial_{\theta }$
11c 17	*F* = *H* = *const*. $G=a_{1}r+a_{2}$ where $a_{1}\text{≠}0$	$\xi_{\left(3\right)}=t\partial_{t}+\frac{a_{1}r+a_{2}}{a_{1}}\partial_{r}+z\partial_{z}$
		$\xi_{\left(4\right)}=\partial_{t}$
		$\xi_{\left(5\right)}=z\partial_{t}+t\partial_{z}$
		$\xi_{\left(6\right)}=-z\cos(a_{1}\theta )\partial_{r}+\frac{z\sin(a_{1}\theta )}{G(r)}\partial_{\theta }+\frac{G(r)\cos(a_{1}\theta )}{a_{1}}\partial_{z}$
		$\xi_{\left(7\right)}=-z\sin(a_{1}\theta )\partial_{r}-\frac{z\cos(a_{1}\theta )}{G(r)}\partial_{\theta }+\frac{G(r)\sin(a_{1}\theta )}{a_{1}}\partial_{z}$
		$\xi_{\left(8\right)}=\cos(a_{1}\theta )\partial_{r}-\frac{\sin(a_{1}\theta )}{G(r)}\partial_{\theta }$
		$\xi_{\left(9\right)}=\sin(a_{1}\theta )\partial_{r}+\frac{\cos(a_{1}\theta )}{G(r)}\partial_{\theta }$
		$\xi_{\left(10\right)}=\frac{G(r)\cos(a_{1}\theta )}{a_{1}}\partial_{t}+\cos(a_{1}\theta )t\partial_{r}-\frac{\sin(a_{1}\theta )t}{G(r)}\partial_{\theta }$
		$\xi_{\left(11\right)}=\frac{G(r)\sin(a_{1}\theta )}{a_{1}}\partial_{t}+\sin(a_{1}\theta )t\partial_{r}+\frac{\cos(a_{1}\theta )t}{G(r)}\partial_{\theta }$
11d 21	*F* = *const*. $G=a_{1}t+a_{2}$ $H=a_{3}r+a_{4}$ where $a_{1}\text{≠}0$ and $a_{3}\text{≠}0$	$\xi_{\left(3\right)}=\frac{a_{1}t+a_{2}}{a_{1}}\partial_{t}+\frac{a_{3}r+a_{4}}{a_{3}}\partial_{r}$
		$\xi_{\left(4\right)}=a_{3}\cos(a_{3}z)\partial_{r}-\frac{a_{3}}{H(r)}\sin(a_{3}z)\partial_{z}$
		$\xi_{\left(5\right)}=a_{3}\sin(a_{3}z)\partial_{r}+\frac{a_{3}}{H(r)}\cos(a_{3}z)\partial_{z}$
		$\xi_{\left(6\right)}=e^{-a_{1}\theta }\partial_{t}+\frac{e^{-a_{1}\theta }}{G(t)}\partial_{\theta }$
		$\xi_{\left(7\right)}=e^{a_{1}\theta }\partial_{t}-\frac{e^{a_{1}\theta }}{G(t)}\partial_{\theta }$
		$\xi_{\left(8\right)}=e^{-a_{1}\theta }[a_{1}H(r)\cos(a_{3}z)\partial_{t}+a_{3}G(t)\cos(a_{3}z)\partial_{r}+\frac{a_{1}H(r)\cos(a_{3}z)}{G(t)}\partial_{\theta }-\frac{a_{3}G(t)\sin(a_{3}z)}{H(r)}\partial_{z}]$
		$\xi_{\left(9\right)}=e^{-a_{1}\theta }[a_{1}H(r)\sin(a_{3}z)\partial_{t}+a_{3}G(t)\sin(a_{3}z)\partial_{r}+\frac{a_{1}H(r)\sin(a_{3}z)}{G(t)}\partial_{\theta }-\frac{a_{3}G(t)\cos(a_{3}z)}{H(r)}\partial_{z}]$
		$\xi_{\left(10\right)}=e^{a_{1}\theta }[a_{1}H(r)\cos(a_{3}z)\partial_{t}+a_{3}G(t)\cos(a_{3}z)\partial_{r}-\frac{a_{1}H(r)\cos(a_{3}z)}{G(t)}\partial_{\theta }-\frac{a_{3}G(t)\sin(a_{3}z)}{H(r)}\partial_{z}]$
		$\xi_{\left(11\right)}=e^{a_{1}\theta }[a_{1}H(r)\sin(a_{3}z)\partial_{t}+a_{3}G(t)\sin(a_{3}z)\partial_{r}-\frac{a_{1}H(r)\sin(a_{3}z)}{G(t)}\partial_{\theta }-\frac{a_{3}G(t)\cos(a_{3}z)}{H(r)}\partial_{z}]$
11e 23	*F* = *H* = *const*. $G=a_{1}t+a_{2}$ where $a_{1}\text{≠}0$	$\xi_{\left(3\right)}=\frac{a_{1}t+a_{2}}{a_{1}}\partial_{t}+r\partial_{r}+z\partial_{z}$
		$\xi_{\left(4\right)}=a_{1}re^{a_{1}\theta }\partial_{t}+G(t)e^{a_{1}\theta }\partial_{r}-\frac{a_{1}re^{a_{1}\theta }}{G(t)}\partial_{\theta }$
		$\xi_{\left(5\right)}=a_{1}re^{-a_{1}\theta }\partial_{t}+G(t)e^{-a_{1}\theta }\partial_{r}-\frac{-a_{1}re^{a_{1}\theta }}{G(t)}\partial_{\theta }$
		$\xi_{\left(6\right)}=a_{1}ze^{a_{1}\theta }\partial_{t}-\frac{a_{1}ze^{a_{1}\theta }}{G(t)}\partial_{\theta }+G(t)e^{a_{1}\theta }\partial_{z}$
		$\xi_{\left(7\right)}=a_{1}ze^{-a_{1}\theta }\partial_{t}-\frac{a_{1}ze^{-a_{1}\theta }}{G(t)}\partial_{\theta }+G(t)e^{-a_{1}\theta }\partial_{z}$
		$\xi_{\left(8\right)}=e^{a_{1}\theta }\partial_{t}-\frac{a_{1}e^{a_{1}\theta }}{G(t)}\partial_{\theta }$
		$\xi_{\left(9\right)}=e^{-a_{1}\theta }\partial_{t}-\frac{a_{1}e^{-a_{1}\theta }}{G(t)}\partial_{\theta }$
		$\xi_{\left(10\right)}=z\partial_{r}-r\partial_{z}$
		$\xi_{\left(11\right)}=\partial_{r}$

**Table 4:** Classification of HVFs.

Metric No./ Branch No.	Metric Coefficients	Proper HVF / Additional KVFs
11f 24	*F* = *F*(*t*) *G* = *const*. $H=a_{1}r+a_{2}$ where $F_{,t}(t)\text{≠}0$ and $a_{1}\text{≠}0$	$\xi_{\left(3\right)}=\frac{\int F(t)dt}{F(t)}\partial_{t}+\frac{a_{1}r+a_{2}}{a_{1}}\partial_{r}+\theta \partial_{\theta }$
		$\xi_{\left(4\right)}=\frac{1}{F(t)}\;\partial_{t}$
		$\xi_{\left(5\right)}=\frac{\theta }{F(t)}\;\partial_{t}+\int F(t)dt\;\partial_{\theta }$
		$\xi_{\left(6\right)}=-a_{1}\theta \sin(a_{1}z)\partial_{r}+H(r)\sin(a_{1}z)\partial_{\theta }-\frac{a_{1}\theta \cos(a_{1}z)}{H(r)}\partial_{z}$
		$\xi_{\left(7\right)}=-a_{1}\theta \cos(a_{1}z)\partial_{r}+H(r)\cos(a_{1}z)\partial_{\theta }+\frac{a_{1}\theta \sin(a_{1}z)}{H(r)}\partial_{z}$
		$\xi_{\left(8\right)}=-a_{1}\sin(a_{1}z)\partial_{r}-\frac{a_{1}\cos(a_{1}z)}{H(r)}\partial_{z}$
		$\xi_{\left(9\right)}=-a_{1}\cos(a_{1}z)\partial_{r}+\frac{a_{1}\sin(a_{1}z)}{H(r)}\partial_{z}$
		$\xi_{\left(10\right)}=\frac{\cos(a_{1}z)}{F(t)}\partial_{t}+a_{1}\cos(a_{1}z)\int F(t)dt\;\partial_{r}-\frac{a_{1}\sin(a_{1}z)\int F(t)dt}{H(r)}\partial_{z}$
		$\xi_{\left(11\right)}=\frac{\sin(a_{1}z)}{F(t)}\partial_{t}+a_{1}\sin(a_{1}z)\int F(t)dt\;\partial_{r}+\frac{a_{1}\cos(a_{1}z)\int F(t)dt}{H(r)}\partial_{z}$
11g 32	*F* = *F*(*t*) *G* = *const*., *H* = *H*(*t*) and *F*(*t*)=*H*,_*t*_(*t*)	$\xi_{\left(3\right)}=\frac{\int F(t)dt}{F(t)}\;\partial_{t}+r\partial_{r}+\theta \partial_{\theta }$
		$\xi_{\left(4\right)}=\frac{\theta e^{z}}{F(t)}\partial_{t}+H(t)e^{z}\partial_{\theta }-\frac{\theta e^{z}}{H(t)}\partial_{z}$
		$\xi_{\left(5\right)}=\frac{\theta e^{-z}}{F(t)}\partial_{t}+H(t)e^{-z}\partial_{\theta }+\frac{\theta e^{-z}}{H(t)}\partial_{z}$
		$\xi_{\left(6\right)}=\frac{re^{z}}{F(t)}\partial_{t}+H(t)e^{z}\partial_{r}-\frac{re^{z}}{H(t)}\partial_{z}$
		$\xi_{\left(7\right)}=\frac{re^{-z}}{F(t)}\partial_{t}+H(t)e^{-z}\partial_{r}+\frac{re^{-z}}{H(t)}\partial_{z}$
		$\xi_{\left(8\right)}=\frac{e^{z}}{F(t)}\partial_{t}-\frac{e^{z}}{\int F(t)dt}\;\partial_{z}$
		$\xi_{\left(9\right)}=-\frac{e^{-z}}{F(t)}\partial_{t}-\frac{e^{-z}}{\int F(t)dt}\;\partial_{z}$
		$\xi_{\left(10\right)}=\theta \partial_{r}-r\partial_{\theta }$
		$\xi_{\left(11\right)}=\partial_{r}$
11h 37	$F=a_{1}t+a_{2}r$ *G* = *H* = *const*., where $a_{1}\text{≠}0$	$\xi_{\left(3\right)}=\frac{a_{1}}{a_{2}^{2}(a_{1}t+a_{2}r)}\partial_{t}+\frac{a_{1}t+a_{2}r}{a_{2}}\partial_{r}+\theta \partial_{\theta }+z\partial_{z}$
		$\xi_{\left(4\right)}=\frac{\theta e^{a_{2}t}}{F(t,r)}\partial_{t}-\theta e^{a_{2}t}\partial_{r}+\int F(t,r)e^{a_{2}t}dt\partial_{\theta }$
		$\xi_{\left(5\right)}=\frac{\theta e^{-a_{2}t}}{F(t,r)}\partial_{t}+\theta e^{-a_{2}t}\partial_{r}+\int F(t,r)e^{-a_{2}t}dt\partial_{\theta }$
		$\xi_{\left(6\right)}=\frac{ze^{a_{2}t}}{F(t,r)}\partial_{t}-ze^{a_{2}t}\partial_{r}+\int F(t,r)e^{a_{2}t}dt\partial_{z}$
		$\xi_{\left(7\right)}=\frac{ze^{-a_{2}t}}{F(t,r)}\partial_{t}+ze^{-a_{2}t}\partial_{r}+\int F(t,r)e^{-a_{2}t}dt\partial_{z}$
		$\xi_{\left(8\right)}=\frac{e^{a_{2}t}}{a_{2}F(t,r)}\partial_{t}-\frac{e^{a_{2}t}}{a_{2}}\partial_{r}$
		$\xi_{\left(9\right)}=\frac{e^{-a_{2}t}}{a_{2}F(t,r)}\partial_{t}-\frac{e^{-a_{2}t}}{a_{2}}\partial_{r}$
		$\xi_{\left(10\right)}=a_{2}\partial_{t}-a_{1}\partial_{r}$
		$\xi_{\left(11\right)}=z\partial_{\theta }-\theta \partial_{z}$
11i 38	$F=a_{1}r+a_{2}$ *G* = *H* = *const*., where $a_{1}\text{≠}0$	$\xi_{\left(3\right)}=\frac{a_{1}r+a_{2}}{a_{1}}\partial_{r}+\theta \partial_{\theta }+z\partial_{z}$
		$\xi_{\left(4\right)}=\frac{\theta e^{a_{1}t}}{F(r)}\;\partial_{t}-\theta e^{a_{1}t}\partial_{r}+\frac{F(r)e^{a_{1}t}}{a_{1}}\partial_{\theta }$
		$\xi_{\left(5\right)}=-\frac{\theta e^{-a_{1}t}}{F(r)}\;\partial_{t}-\theta e^{-a_{1}t}\partial_{r}+\frac{F(r)e^{-a_{1}t}}{a_{1}}\partial_{\theta }$
		$\xi_{\left(6\right)}=\frac{ze^{a_{1}t}}{F(r)}\;\partial_{t}-ze^{a_{1}t}\partial_{r}+\frac{F(r)e^{a_{1}t}}{a_{1}}\partial_{z}$
		$\xi_{\left(7\right)}=-\frac{ze^{-a_{1}t}}{F(r)}\;\partial_{t}-ze^{-a_{1}t}\partial_{r}+\frac{F(r)e^{-a_{1}t}}{a_{1}}\partial_{z}$
		$\xi_{\left(8\right)}=-\frac{e^{a_{1}t}}{F(r)}\;\partial_{t}+e^{a_{1}t}\partial_{r}$
		$\xi_{\left(9\right)}=\frac{e^{-a_{1}t}}{F(r)}\;\partial_{t}+e^{-a_{1}t}\partial_{r}$
		$\xi_{\left(10\right)}=z\partial_{\theta }-\theta \partial_{z}$
		$\xi_{\left(11\right)}=\partial_{t}$

The number of homothetic symmetries in different cases gives information about how restricted the spacetime is. Spacetimes possessing only three HVFs are the most general, that can change with time and are highly anisotropic in nature. A special class of spacetimes is that admitting four or five HVFs, which often have plane symmetry, power-law expansion, or wave-like behavior. The spacetimes with eleven homothetic symmetries are the most symmetric of all. These spacetimes have constant curvature and are just flat Minkowski space, de Sitter space, or anti-de Sitter space written in unusual coordinates. Hence, for increasing number of symmetries, the spacetime becomes more isotropic and geometrically important.

## 3. Solution of the field equations

In this section, we solve the EFEs for the metrics presented in the previous section by considering an imperfect fluid source of matter with stress-energy tensor given by [23]:


$$\begin{array}{c} T_{mn}=(\rho +p)u_{m}u_{n}+pg_{mn}+(p_{r}-p)\chi_{m}\chi_{n}+(p_{\theta }-p)\eta_{m}\eta_{n}+(p_{z}-p)\zeta_{m}\zeta_{n}. \end{array}$$ (3.1)

In the above expression, ρ represents the energy density, the directional pressures are denoted by $p_{r},p_{\theta },$ and $p_{z},$ while $p=\frac{p_{r}+p_{\theta }+p_{z}}{3}$ signifies the isotropic pressure. The energy density is measured by a comoving observer with a timelike four-velocity vector $u^{m}$ satisfying the condition $u^{m}u_{m}=-1.$ Moreover, $\chi^{m},\eta^{m},$ and $\zeta^{m}$ are spacelike vectors satisfying the conditions $\chi^{m}\chi_{m}=\eta^{m}\eta_{m}=\zeta^{m}\zeta_{m}=1$ and $\chi^{m}\eta_{m}=\chi^{m}\zeta_{m}=\eta^{m}\zeta_{m}=\chi^{m}u_{m}=\eta^{m}u_{m}=\zeta^{m}u_{m}=0.$ If $p_{r}=p_{\theta }=p_{z}=p,$ equation (3.1) is reduced to the simpler form $T_{mn}=(\rho +p)u_{m}u_{n}+pg_{mn},$ representing a perfect fluid. Furthermore, the equations $w_{1}=\frac{p_{r}(t,r)}{\rho (t,r)},w_{2}=\frac{p_{\theta }(t,r)}{\rho (t,r)}$ and $w_{3}=\frac{p_{z}(t,r)}{\rho (t,r)},$ respectively define the directional equations of state, while the average equation of state is given by $w=\frac{p(t,r)}{\rho (t,r)}=\frac{1}{3}(w_{1}+w_{2}+w_{3}).$

In the framework of the considered metric (2.1), the terms $u^{m},$ $\chi^{m},\eta^{m},$ and $\zeta^{m}$ are respectively defined as $u^{m}=(\frac{1}{F},0,0,0),$ $\chi^{m}=(0,1,0,0),$ $\eta^{m}=(0,0,\frac{1}{G},0)$ and $\zeta^{m}=(0,0,0,\frac{1}{H}).$ Moreover, for an imperfect fluid source, the EFEs give rise to the following five equations.


$$\begin{array}{c} \frac{G_{,t}H_{,t}}{F^{2}GH}-\frac{G_{,rr}}{G}-\frac{H_{,rr}}{H}-\frac{G_{,r}H_{,r}}{GH}=\rho \end{array}$$ (3.2)

$$\begin{array}{c} \frac{G_{,r}H_{,r}}{GH}+\frac{F_{,r}H_{,r}}{FH}+\frac{F_{,r}G_{,r}}{FG}-\frac{G_{,tt}}{F^{2}G}-\frac{H_{,tt}}{F^{2}H}-\frac{G_{,t}H_{,t}}{F^{2}GH}+\frac{F_{,t}H_{,t}}{F^{3}H}+\frac{F_{,t}G_{,t}}{F^{3}G}=p_{r} \end{array}$$ (3.3)

$$\begin{array}{c} \frac{F_{,rr}}{F}+\frac{H_{,rr}}{H}+\frac{F_{,r}H_{,r}}{FH}-\frac{H_{,tt}}{F^{2}H}+\frac{F_{,t}H_{,t}}{F^{3}H}=p_{\theta } \end{array}$$ (3.4)

$$\begin{array}{c} \frac{F_{,rr}}{F}+\frac{G_{,rr}}{G}+\frac{F_{,r}G_{,r}}{FG}-\frac{G_{,tt}}{F^{2}G}+\frac{F_{,t}G_{,t}}{F^{3}G}=p_{z} \end{array}$$ (3.5)

$$\begin{array}{c} FHG_{,tr}+FGH_{,tr}-GF_{,r}H_{,t}-HF_{,r}G_{,t}=0 \end{array}$$ (3.6)

Out of the above field equations, equation (3.6) arises from the off-diagonal tr−component of $T_{mn}$ and it acts as a necessary condition for a metric to represent an imperfect fluid solution. The metrics violating equation (3.6) correspond to other matter sources. For all metrics satisfying equation (3.6), the remainning four field equations (3.2)–(3.5) can be solved to find the energy density and directional pressures. The metric coefficients of the models 5*g*, 5*i*, 5*j*, 5*k* and 11a−11i identically satisfy equation (3.6), while the remaining four field equations give $\rho =p_{r}=p_{\theta }=p_{z}=0.$ Thus all these metrics represent vacuum solutions of EFEs. Some of the metrics, namely 3b−3f, 3h−3l, 4a−4d, 4*f* and 5*a*, do not satisfy equation (3.6), showing that these models do not *g*ive imperfect fluid solutions. All the remaining metrics represent imperfect fluid solutions with energy density and directional pressures as presented in Table 5.

**Table 5:** Physical quantities for imperfect fluid solutions.

Metric No.	Energy Density and Directional Pressures
3a	$\rho =\frac{a_{3}(a_{1}-a_{3})}{(a_{1}r+a_{2}{)}^{2}},$ $p_{r}=\frac{a_{5}(a_{1}-a_{5})}{(a_{1}t+a_{4}{)}^{2}},$ $p_{\theta }=p_{r},$ $p_{z}=-\rho$
3g	$\rho =\frac{a_{5}(a_{1}-a_{5})}{(a_{1}r+a_{4}{)}^{2}},$ $p_{r}=\frac{a_{3}(a_{1}-a_{3})}{(a_{1}t+a_{2}{)}^{2}},$ $p_{\theta }=-\rho ,$ $p_{z}=p_{r}$
3l	$\rho =\frac{a_{3}(a_{1}-a_{3})}{(a_{1}r+a_{2}{)}^{2}},$ $p_{r}=p_{\theta }=0,$ $p_{z}=-\rho$
4e	$\rho =-\frac{a_{1}^{2}(a_{3}-2a_{4}{)}^{2}}{a_{3}^{2}(a_{1}r+a_{2}{)}^{2}},$ $p_{r}=\frac{a_{1}^{2}(a_{3}-2a_{4})}{a_{3}(a_{1}r+a_{2}{)}^{2}},$ $p_{\theta }=-\frac{2a_{1}^{2}a_{4}(a_{3}-2a_{4})}{a_{3}^{2}(a_{1}r+a_{2}{)}^{2}},$ $p_{z}=0$
4g	$\rho =p_{\theta }=0,$ $p_{r}=-\frac{2a_{3}(a_{1}-2a_{3})}{(a_{1}t+2a_{2}{)}^{2}},$ $p_{z}=-p_{r}$
5b	$\rho =p_{z}=0,$ $p_{r}=p_{\theta }=\frac{2a_{5}(a_{3}-2a_{5})}{(a_{3}t+2a_{4}{)}^{2}}$
5c	$\rho =p_{\theta }=0,$ $p_{r}=p_{z}=\frac{2a_{3}(a_{1}-2a_{3})}{F^{2}(a_{1}t+2a_{2}{)}^{2}}+\frac{F^{\prime }(a_{1}-2a_{3})}{F^{3}(a_{1}t+2a_{2})}$
5d	$\rho =p_{\theta }=0,$ $p_{r}=-\frac{2a_{3}(a_{1}-2a_{3})}{(a_{1}t+2a_{2}{)}^{2}},$ $p_{z}=-p_{r}$
5e	$\rho =\frac{2a_{5}(a_{3}-2a_{5})}{(a_{3}r+2a_{4}{)}^{2}},$ $p_{r}=p_{z}=0,$ $p_{\theta }=-\rho$
5f	$\rho =\frac{2a_{3}(a_{1}-2a_{3})}{(a_{1}r+2a_{2}{)}^{2}},$ $p_{r}=p_{z}=0,$ $p_{\theta }=-\rho$
5h	$\rho =p_{r}=0,$ $p_{\theta }=p_{z}=-\frac{2a_{3}(a_{1}-2a_{3})}{(a_{1}r+2a_{2}{)}^{2}}$

It is important to mention here that the metrics that do not represent imperfect fluid solutions still remain physically relevant. The failure of imperfect fluid condition just indicates that these metrics represent some other classes of matter sources, for example heat flow, null radiation, viscosity, or more general anisotropic energy-momentum tensors. Consequently, these metrics may provide useful solutions for alternative physical scenarios beyond the imperfect fluid framework, adopted in the current study.

For the metrics 3*l*, 5*e* and 5*f*, the energy density is non-vanishing, pressure in one direction coincides with −ρ, while the other two directional pressures are zero. These models represent highly anisotropic fluids for which one spatial direction behaves like a vacuum energy component having the directional equation of state w=−1, while the other two directions behave dust-like with *w* = 0. For models 4*g*, 5*b*, 5*c*, 5*d* and 5*h*, two directional pressures are non-zero while the third directional pressure and energy density vanish. The directional equations of state $w_{i}$ are ill-defined for such models. However, the spacetimes still carry effective stresses. The remaining three metrics (labeled 3*a*, 3*g* and 4*e*) represent fully anisotropic fluid confi*g*urations. For each of these metrics, the energy density is non-zero and the directional pressures are non-uniform. For the models 3*a* and 3*g*, one directional pressure is same as −ρ, which indicates the presence of a vacuum-like component along a spatial direction. For both these metrics, the remaining two directions exhibit equal fluid pressures. These type of confi*g*urations are analogous to anisotropic dark-energy type fluids and may serve as effective descriptions of cylindrically symmetric sources with internal tension or strin*g*-like behavior. Finally, for the metric 4*e*, all the three directional pressures are different and one of them becomes zero, which represents a mixture of vacuum-like, fluid-like, and dust-like behavior within a single spacetime. These features highlight the ability of non-static cylindrically symmetric geometries to accommodate highly non-trivial anisotropic matter distributions.

### 3.1. Energy conditions

In order to assess the physical plausibility of the obtained models, here we discuss the positivity of energy density, and different energy conditions (weak (WEC), strong (SEC) and dominant (DEC)). For an imperfect fluid with energy-momentum tensor of the form (3.1), these energy conditions can be checked using the expressions for $\rho ,p_{r},p_{\theta }$ and $p_{z}$ presented in Table 5. For metrics 3*a* and 3*l*, the positivity of ρ requires that the constants *a*_3_ and $a_{1}-a_{3}$ have the same sign. Similarly, for metric 3*g*, the constants *a*_5_ and $a_{1}-a_{5}$ must have the same sign in order to get positive energy density. Fina*ll*y, the metrics 5*f* and 5*h* respectively give positive energy density provided that $a_{5}(a_{3}-2a_{5})\geq 0$ and $a_{3}(a_{1}-2a_{3})\geq 0.$ For the remaining metrics listed in Table 5, energy density either vanishes or it is positive, leadin*g* to physically realistic models.

For an imperfect fluid, the WEC requires $\rho \geq 0,\rho +p_{r}\geq 0,\rho +p_{\theta }\geq 0$ and $\rho +p_{z}\geq 0.$ The expressions for $\rho ,p_{r},p_{\theta }$ and $p_{z}$ given in Table 5 can be easily used in these inequalities to check the WEC. For example, for metric 3*l*, we have $\rho =\frac{a_{3}(a_{1}-a_{3})}{(a_{1}r+a_{2}{)}^{2}},$ $p_{r}=p_{\theta }=0$ and $p_{z}=-\rho .$ These quantities clearly satisfy the WEC provided that $a_{3}(a_{1}-a_{3})\geq 0,$ giving a neat parameter restriction.

The SEC is expressed by the inequalities $\rho +p_{r}+p_{\theta }+p_{z}\geq 0,$ $\rho +p_{r}\geq 0,\rho +p_{\theta }\geq 0$ and $\rho +p_{z}\geq 0.$ For the previously mentioned metric 3*l*, we have $\rho +p_{r}+p_{\theta }+p_{z}=0$ and the remaining three inequalities give $a_{3}(a_{1}-a_{3})\geq 0.$ Thus both weak and strong energy conditions are satisfied under the same restriction $a_{3}(a_{1}-a_{3})\geq 0$ on the parameters involved.

Finally, the dominant energy condition requires ρ≥0, $\rho \geq |p_{r}|,$ $\rho \geq |p_{\theta }|,$ and $\rho \geq |p_{z}|.$ One can easily see that the same restriction $a_{3}(a_{1}-a_{3})\geq 0$ on the parameters *a*_1_ and *a*_3_ ensures that DEC is also satisfied for the metric 3*l*.

For all other metrics listed in Table 5, one can easily check the WEC, SEC and DEC in a similar way.

### 3.2. Graphical behavior of physical quantities

The physical properties of the derived imperfect fluid solutions can be further studied by illustrating the energy density and directional pressures graphically. From Table 5, it is clear that energy density and directional pressures for the obtained solutions depend on several arbitrary constants. Here we choose some specific parameter values for a representative metric 3*a* for which the energy density remains non-negative and draw the graphs of the resulting expressions for energy density and directional pressures. The purpose of these plots is to illustrate the qualitative behavior of these variables and to highlight the effects of anisotropy inherent in the obtained solution.

**Fig 2:**
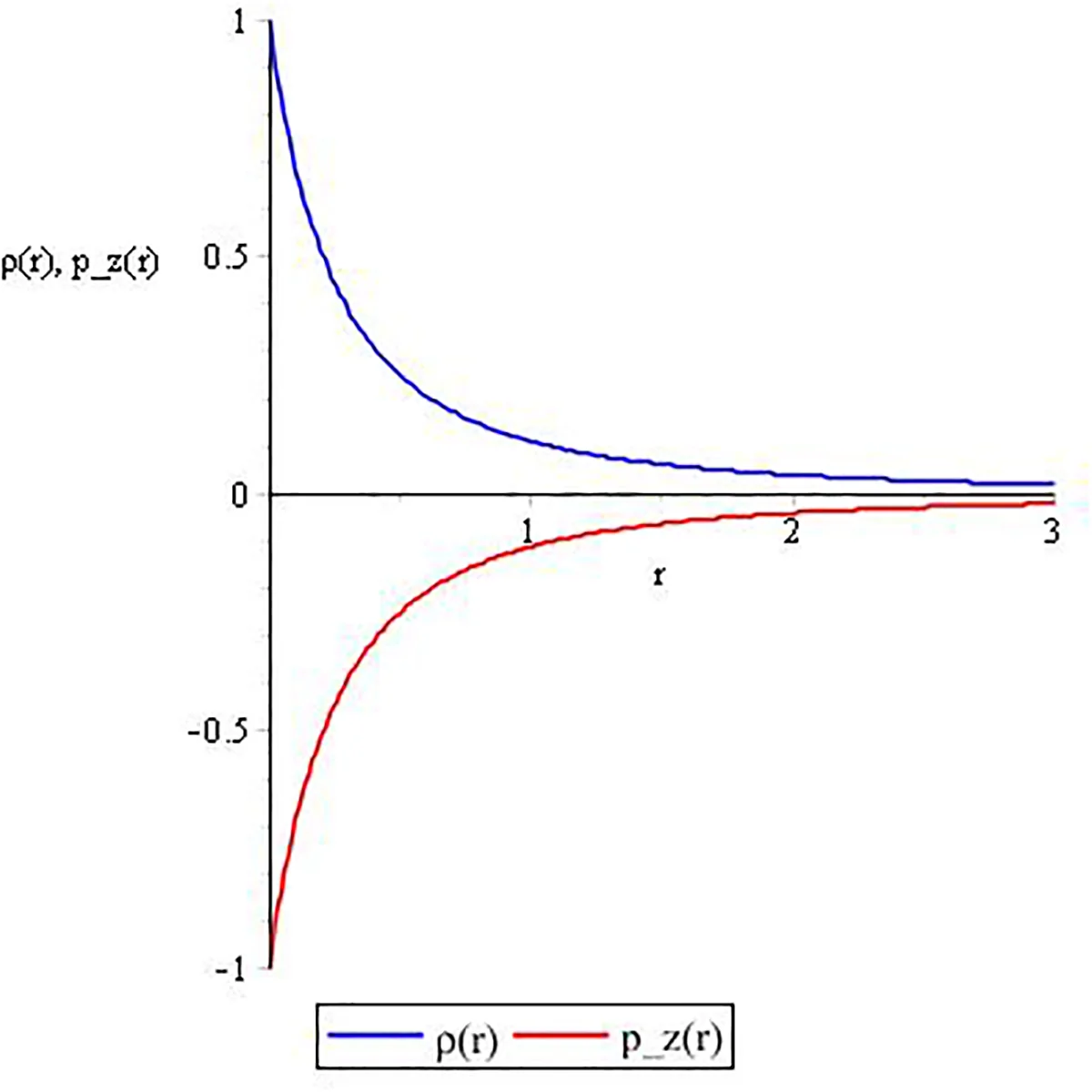
Graphical behavior of ρ and $p_{z}$ for metric 3*a* with *a*_1_ = 2 and $a_{2}=a_{3}=a_{4}=a_{5}=1$.

For the metric 3*a*, we chose *a*_1_ = 2 and $a_{2}=a_{3}=a_{4}=a_{5}=1,$ so that the corresponding energy density and directional pressures become $\rho =\frac{1}{(2r+1{)}^{2}},$ $p_{r}=p_{\theta }=\frac{1}{(2t+1{)}^{2}}$ and $p_{z}=-\frac{1}{(2r+1{)}^{2}}.$ Both ρ and the longitudinal pressure $p_{z}$ depend on the radial coordinate *r* and their graphical behavior is shown in Fig 2. It is observed that for any value of *r*, the energy density ρ remains positive, and with increasing radial distance it decreases monotonically. On the other hand, the longitudinal pressure $p_{z}$ exhibits the same magnitude but opposite sign, which indicates the presence of tension along the symmetry axis. For large values of *r*, both ρ and $p_{z}$ approach zero, which shows that the matter distribution becomes progressively diluted away from the axis.

**Fig 3:**
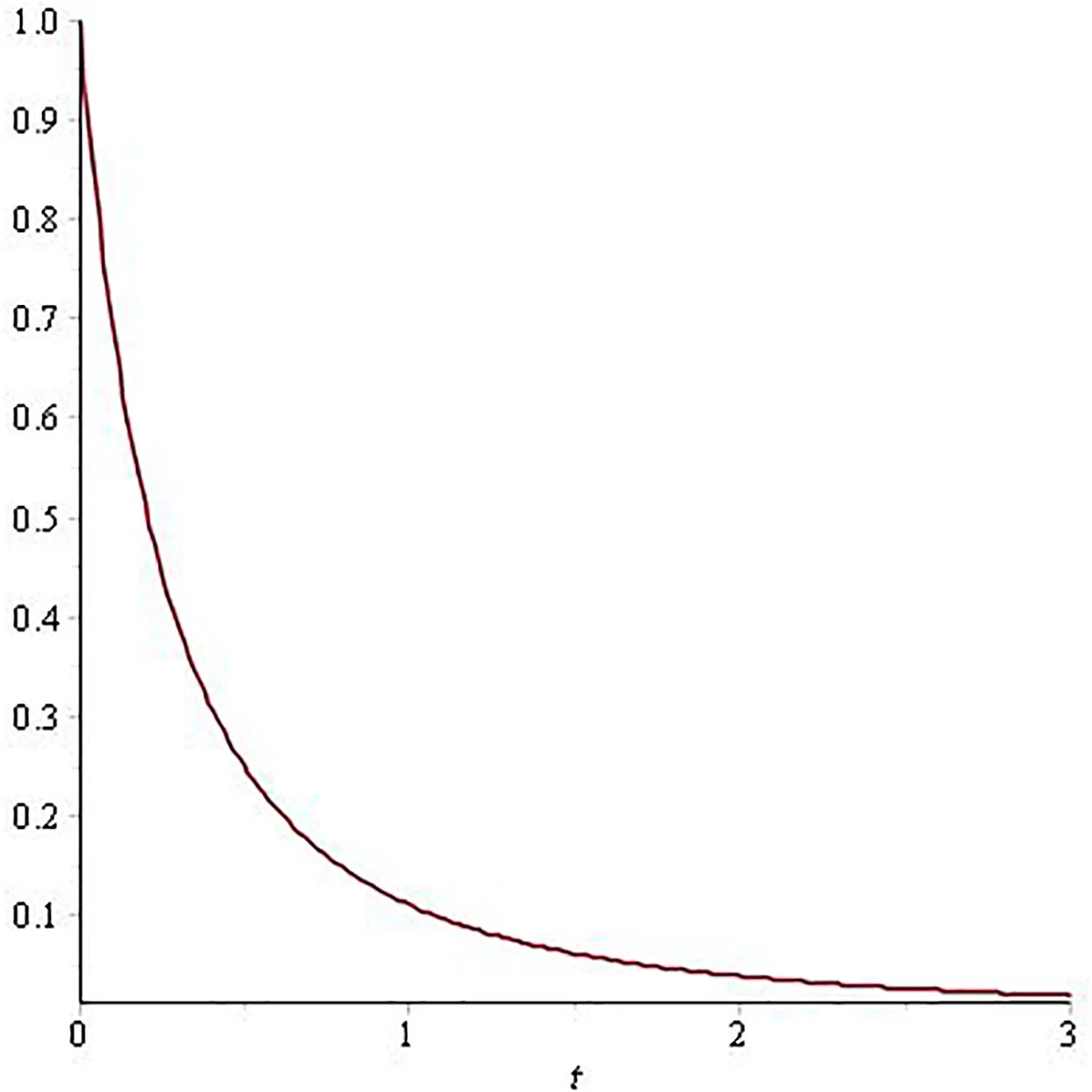
Graphical behavior of $p_{r}$ and $p_{\theta }$ for metric 3*a* with *a*_1_ = 2 and $a_{2}=a_{3}=a_{4}=a_{5}=1$.

The evolution of the radial and azimuthal pressures is illustrated in Fig 3. As both these pressures are identical functions of time, the corresponding curves coincide. Moreover, both the pressures are positive and decrease monotonically with increasing time, approaching zero asymptotically. This behavior of the pressure in two directions shows that the fluid gradually loses pressure support as the spacetime evolves while the isotropy in the transverse directions is maintained.

It is worth mentioning here that both ρ and $p_{z}$ for metric 3*a* become singular when $a_{1}r+a_{2}=0.$ Similarly, for $a_{1}t+a_{4}=0,$ the transverse pressures become singular. For the above chosen parameter values, these singularities occur outside the plotted domain. Away from these singular hypersurfaces, the matter variables remain finite and exhibit a smooth monotonic decay, tending to zero for large values of *r* and *t*.

## 4. Connection to known models in the literature

In this section, we relate the derived metrics of the present classification with some well known cylindrically symmetric models that have been widely studied in the literature. This comparison ensures the correctness of our results as well as clarifies their physical and geometrical significance.

Some of our derived metrics exhibit the time-dependent characteristic of cylindrical gravitational waves. For example in case of the metrics 5*i* and 5*j*, the dependence of the metric functions on *t* and *r* is consistent with wave-like cylindrical geometries. For some specific values of the parameters involved, the metric coefficients of some of our derived metrics (for example 4*e* and 5*g*) are linear functions of *r* only. These metrics reduce to the Levi-Civita spacetime representing static gravitational field of an infinitely long line mass. The metric 4*f* also shares the Levi-Civita radial from, however because of the involvement of the term $e^{t}$ in one of the metric coefficients, it can be considered as simple time-dependent extension of the static solution. For the metrics 4*b*, 4*c*, 4*d*, 5*h*, 11*h*, and 11*i*, the two transverse metric coefficients *G* and *H* become equal due to which the (θ,z) sector becomes isotropic and the spacetime admits the rotational Killing symmetry $z\partial_{\theta }-\theta \partial_{z},$ a defining feature of plane symmetry. The reduction of the mentioned metrics to the plane symmetric models establishes a link between our derived results and the well known plane symmetric solutions. The metric coefficients of the models 4*g*, 5*c* and 5*d* involve the term $(a_{1}t+2a_{2}{)}^{(1-2a_{3})/a_{1}},$ which leads to anisotropic power-law scalings in the transverse directions. Due to this feature, these models resemble the behavior of Kasner-type solutions, a standard reference model for anisotropic expansion in general relativity. These metrics show the essential feature of Kasner-type solutions: expansion in some directions and contraction in others. However, in cylindrical coordinates, this behavior appears with one non-changing direction. For two models, labeled 5*i* and 5*j*, one of the metric functions depends on *t* + *r*, which represents a null or boost-aligned structure. For these metrics, the existence of the Killin*g* symmetry $\partial_{r}-\partial_{t}$ shows that the geometry remains unchanged along surfaces of constan*t t* + *r*, which is a feature of self-similar wavefronts propagating along the symmetry axis and is nearly related to similarity solutions and traveling-front models that occur in cylindrical wave spacetimes. Finally, the metrics 11a−11i admitting eleven symmetries correspond *t*o maximally symmetric spacetimes. Thus the present classification not only produce non-static cylindrical and wave-like solutions, as limiting cases it also recovers the maximally symmetric spacetimes.

In Table 6, we present some explicit parameter choices for which the derived metrics in the current study reduce to the well-known cylindrically and plane symmetric solutions.

**Table 6:** Connection of the derived metrics with known solutions.

Metric	Parameter Choices	Properties /Resulting Metric	Geometric Interpretation
4*b*	Any parameters	*G* = *H*	Plane symmetric geometry with isotropic transverse sector
4*c*	Any parameters	*G* = *H*	Plane symmetric cosmological type geometry
4*d*	Any parameters	*G* = *H*	Self-similar plane symmetric geometry
4*e*	$a_{2}=a_{4}=0,a_{3}=1$	Metric functions become linear in *r*	Static cylindrically symmetric geometry with Levi-Civita type radial behavior
4*f*	$a_{1}=1,a_{2}=0$	Linear radial dependence together with an exponential time factor	Time-dependent extension of a cylindrically symmetric geometry
4*g*	$a_{1}=a_{3}=1,a_{2}=0,$ *F* = *H* = 1	$ds^{2}=-dt^{2}+dr^{2}+t^{-2}d\theta^{2}+dz^{2}$	Kasner-like anisotropic power-law behavior
5*c*	$a_{1}=1,a_{2}=0,$ *F* = 1	Power-law evolution in one transverse direction	Kasner-like anisotropic evolution
5*d*	$a_{1}=1,a_{2}=0$	Power-law evolution in one transverse direction	Kasner-like anisotropic evolution
5*g*	$a_{1}=1,a_{2}=0,$ *F* = *G* = 1	$ds^{2}=-dt^{2}+dr^{2}+d\theta^{2}+r^{2}dz^{2}$	Static cylindrically symmetric metric with linear radial scale factor
5*i*, 5*j*	*a*_1_ = 1	Metric functions depend on *t* + *r*	Cylindrical wave-like geometries

Though the primary goal of the present study is to classify non-static cylindrically symmetric spacetimes via HVFs, the derived solutions possess certain properties that may be relevant in physical applications. The metrics which exhibit an anisotropic power-law evolution may serve as suitable models of anisotropic cosmological expansion. The models admitting wave-like structures may be pivotal in studying cylindrical gravitational waves. Several of the derived solutions are plane symmetric, which give geometrical backgrounds for the investigation of anisotropic matter distributions and symmetry-restricted cosmological models. Similarly, the obtained vacuum solutions may be used as exact backgrounds for the study of test particle motion. Hence the solutions obtained in the current study yield a broad framework for future investigations of physically motivated relativistic systems.

## 5. Conclusion

In this paper, we have used a powerful computational tool (Rif tree approach) to systematically investigate HVFs of non-static cylindrical spacetime. The classification led to an extensive collection of exact solutions, classified by their symmetry content into families with 3, 4, 5 and 11 symmetries, including proper HVFs and additional KVFs. In Ref. [24], the authors obtained only four HVFs with one proper homothety and three KVFs for static version of the cylindrically symmetric metric considered in the present study. The case of three HVFs is not possible in the static case as in that case the spacetime admits at least three KVFs. However, the metrics with five and eleven symmetries derived in the present study of non-static spacetime arise due to the introduction of time dependent scale factors in the considered non-static metric.

The most important aspect of the current study is the role of HVFs in generating self-similar spacetime structures. Homothetic symmetries represent scale invariance of the spacetime geometry and are closely linked with self-similar solutions of EFEs, which play a pivotal role in studying gravitational collapse, critical phenomena and anisotropic cosmological models. For the derived metrics, the existence of proper HVFs indicates that these solutions possess an underlying self-similar structure, which make them potentially useful for the investigation of scale-invariant gravitational processes. Hence the present classification gives a broad family of exact solutions that may serve as useful models in such studies.

We have also solved the EFEs for the derived metrics for an imperfect fluid source and obtained explicit expressions for energy density and directional pressures. The derived solutions exhibit a rich variety of physical behaviors, including vacuum configurations, highly anisotropic fluids with vacuum-like or dust-like components, and wave-like geometries. Out of the derived metrics, many reduce to the well-known solutions in the literature such as Levi-Civita, Kasner-type, plane symmetric, and cylindrical gravitational wave spacetimes, thereby validating our classification and embedding it within the broader landscape of exact solutions in general relativity.

The spacetime models derived in the current classification demonstrate the richness of cylindrically symmetric geometries admitting HVFs. Moreover, the current classification highlights how different combinations of HVFs and KVFs lead to a wide variety of geometrical structures, ranging from minimally symmetric to highly constrained spacetimes. The existence of cylindrically symmetric models possessing large number of symmetries shows that homothetic symmetry provides a powerful organizing principle for constructing and understanding exact solutions. This systematic classification considerably enlarges the set of known cylindrically symmetric geometries and establishes a unified framework for their further mathematical and physical investigation.

Our work demonstrates that homothetic symmetry serves as a powerful organizing principle for constructing and interpreting time-dependent, anisotropic spacetimes. The Rif tree method proved particularly effective in uncovering metrics that might be overlooked by traditional integration techniques, thereby extending the catalog of known cylindrically symmetric solutions.

The present analysis is carried out for an imperfect fluid source, that provides a sufficiently general framework to accommodate a wide variety of matter configurations and anisotropic pressures. However, the derived models in this study do not depend on the chosen matter model and may also serve as geometrical backgrounds for other physically relevant sources, such as electromagnetic, scalar, and viscous fluids. The additional physical implications of the obtained models may be revealed by the investigation of these alternative matter sources. Such an investigation points an interesting direction for future research.

Another possible direction for the extension of the present work is to investigate the dynamical and perturbative stability of the derived solutions. As the present classification yielded a number of distinct metric families with different physical properties, a detailed stability analysis of all these solutions would require separate treatment of each set of solutions and is therefore left for future study.
